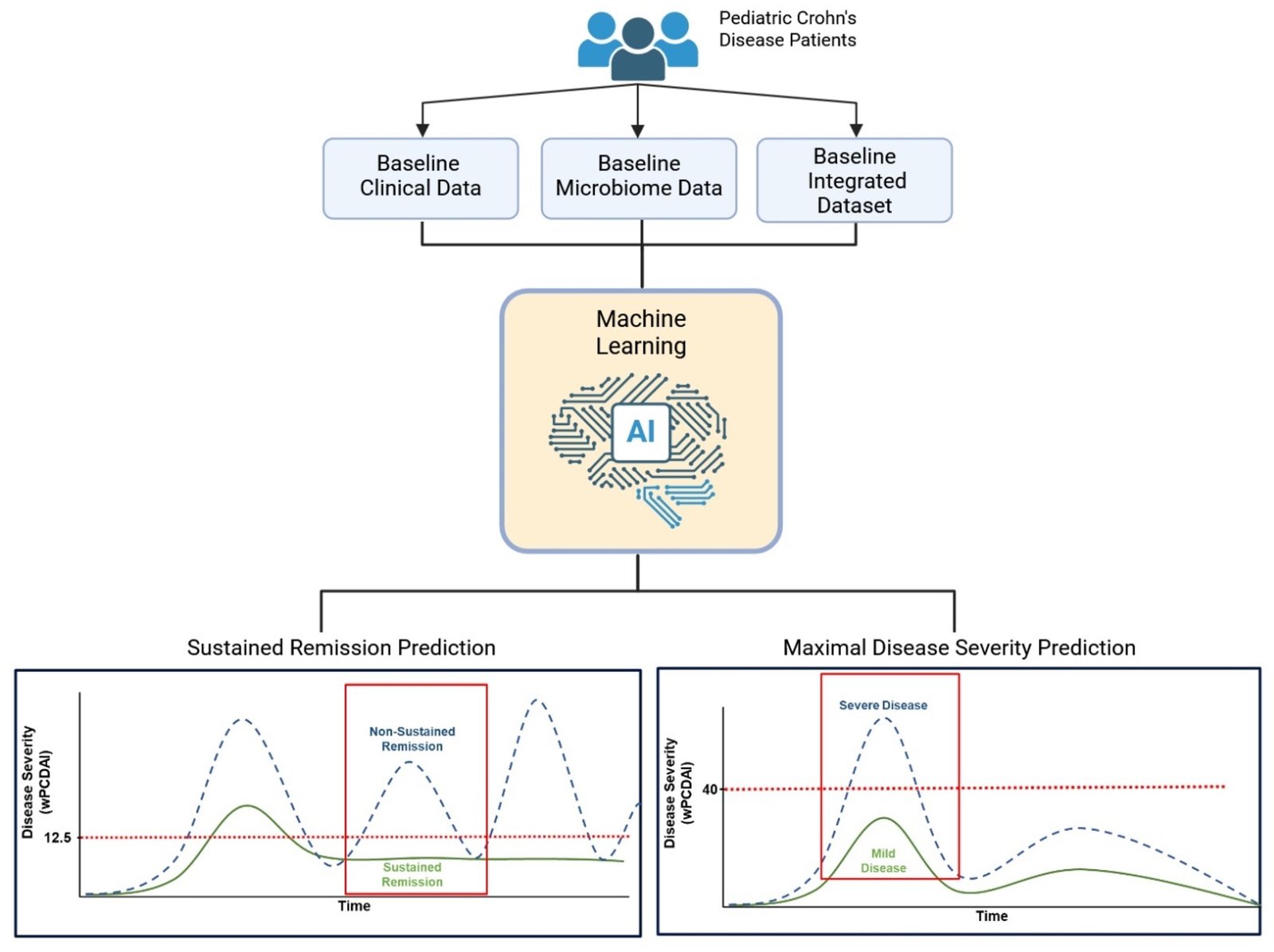# Poster Session II – Poster of Distinction II - A212 PREDICTING SUSTAINED REMISSION AND MAXIMAL DISEASE SEVERITY IN PEDIATRIC CROHN’S DISEASE USING MACHINE LEARNING

**DOI:** 10.1093/jcag/gwaf042.212

**Published:** 2026-02-13

**Authors:** I Ng, H Sham, K Jacobson, K Korthauer, B Vallance

**Affiliations:** Bioinformatics, The University of British Columbia, Vancouver, BC, Canada; BC Children’s Hospital Research Institute, Vancouver, BC, Canada; BC Children’s Hospital, Vancouver, BC, Canada; Bioinformatics, The University of British Columbia, Vancouver, BC, Canada; Bioinformatics, The University of British Columbia, Vancouver, BC, Canada

## Abstract

**Background:**

Pediatric Crohn’s disease (CD) is a chronic inflammatory condition affecting the gastrointestinal tract. It displays more heterogeneous disease trajectories and treatment responses than adult-onset cases, posing significant management challenges. While patients following more severe trajectories may benefit from early aggressive treatments, no reliable objective method exists to identify which children will follow a severe trajectory at diagnosis. This prognostic gap leaves risk stratification dependent on subjective clinical judgment, potentially delaying interventions for high-risk patients. Early identification of severe trajectories could transform treatment decisions and improve outcomes through timely aggressive therapy.

**Aims:**

This study aims to predict one-year sustained remission and maximal disease severity using machine learning models trained on baseline clinical and microbiome data in a nation-wide cohort of Canadian children with CD.

**Methods:**

Using baseline clinical and microbiome data from the Canadian Children IBD Network inception cohort, we developed machine learning models to predict two first-year outcomes: 1) sustained remission vs non-sustained remission, defined as maintaining a post-remission Weighted Pediatric Crohn’s Disease Activity Index (wPCDAI) <12.5 without inflammatory episodes, and 2) maximal disease severity (remission/mild [post-diagnosis wPCDAI <40] vs moderate/severe [wPCDAI ≥40]). Nine algorithms were trained on three data modalities (clinical alone, microbiome alone, and integrated clinical-microbiome) using repeated nested K-fold cross-validation, with minimum redundancy maximal relevance feature selection, Bayesian hyperparameter optimization, and SHAP for model explainability.

**Results:**

For sustained remission prediction, integrated models outperformed microbiome- or clinical-only models, with integrated logistic regression achieving the highest mean AUC (0.763); key features included initial treatment at diagnosis, disease location, and wPCDAI at diagnosis, as well as taxa known to play a role in CD such as *Haemophilus* and *Lachnospiraceae*. For maximal disease severity prediction, microbiome models performed best, with Gaussian naïve Bayes reaching a mean AUC of 0.801 and highlighting microbes such as *Clostridium* and *Veillonella* as predictors of severe disease, while taxa such as *Coprococcus* and *Romboutsia* were associated with milder disease.

**Conclusions:**

Our results demonstrate the potential of integrated machine learning approaches to support clinical decision-making in pediatric Crohn’s disease. By enabling early identification of high-risk patients at diagnosis, this work paves the way for personalized treatment strategies that could improve long-term outcomes in this vulnerable population.

**Funding Agencies:**

CIHRBCCHRI, Government of British Columbia